# The Relative Values of Non Cohesive and Cohesive Gold

**Published:** 1892-06

**Authors:** I. C. Curtis


ARTICLE III.

### THE RELATIVE VALUES OF NON-COHESIVE AND COHESIVE GOLD.

I will preface my few remarks by a quotation from a familiar and popular author on dental practice in his remarks on the use of gold as a filling material.

I refer to Dr. J. Foster Flagg, where he says, "It is a work which can be best done by not more than one worker in a thousand; a work which can be well done by not more than one worker in a hundred; a work which is not reasonably well done by more than one worker in ten, and yet a work which is attempted in gold-working dentistry by nine workmen out of ten."

Happy is the one man in the thousand that can best insert a gold plug. To him these remarks are not addressed, but it is for the benefit of the remaining nine hundred and ninety-nine that this article is written.

As far as is known, with gold as first used, and down to a comparatively recent date, no attempt was made to take advantage of its cohesive properties. In fact, an effort was made by the manufacturers to produce a gold that was uniformly soft.

I think I am correct when I say that to Dr. Robert Arthur is due the credit of bringing prominently before the profession some of the advantages of cohesive over non-cohesive gold. Dr. Arthur published a small volume on the subject in the year 1856, which had at that time a wide circulation, and did much to revolutionize dental practice, and since that time down to a very few years a fierce war has been waged between non cohesive and cohesive workers. Reams of paper and rivers of ink have been used, and the result has been that the conservative worker has selected the poor part from each side, the one class conceding that for restoring contour, cohesive was best, and the other that with frail walls and at the cervical margins non-cohesive gold could be forced into the interstices with less danger of fracture or mechanical disintegration of the margins of the cavity; and to-day many of the best workers use non-cohesive gold at the cervical walls and the body of the cavity, finishing the filling and restoring the contour with cohesive, making a combination that is at times very desirable.

I wish to state here that I do not refer to restoring broken corners or building down teeth, for here cohesive gold is the material that must be used, but do refer in a general way to all other classes and modifications with a few exceptions.

At the risk of being called egotistical, I will explain the plan that has given me the most satisfaction, and resulted in the fewest failures; and as no advice is valuable that is not based on experience, and as we profit most by our failures, let it be assumed at the beginning that I have had my share of failures, and belong to Dr. Flagg's nine hundred and ninety-nine.

For convenience, we will divide the cavities we find into:

1. Small ones the size of a pin's head and a little larger.

2. Those with corners or whole parts to be restored.

3. Proximal cavities in the anterior teeth.

4 Proximal cavities in the bicuspids and molars.

5. Cavities on the labial surfaces of incisors and cuspids, and the buccal surfaces of molars.

In the first and second classes, the almost uniform results have been in favor of cohesive gold through the entire operation, and it is not my province at this time to enter into detail as to its manipulations. Each operator is presumed to have a plan that is peculiarly adapted to himself.

In the third class referred to, namely, those on the proximal surfaces of incisors and cuspids, the plan I have adopted (after separation) is to so shape the cavity so as to have an undercut at both extremities of it; beveling both lingual and labial edges, not depending on the last two for the retention of the plug to be inserted. After the cavity is prepared, I take the proper size piece of No. 4 soft foil, and fold it into a ribbon that is one-third wider than the long diameter of the cavity; then take a small instrument, say a broken excavator, and roll into a cyclinder (do not roll too closely). Experience will tell you how much gold you will require for the cylinder. It will vary from one-fifth to a whole sheet. (I prefer these cylinders to any found in the depots.)

It is presumed the dam is applied. You will then insert the cylinder into the cavity, forcing it up at the cervical margin, and allow it to protrude both at the cervical and lingual margins, forcing it into the undercuts at each end of the cavity, making a sort of basket of gold in your cavity; then begin with cohesive gold at the cervical, and finish the cavity with it, and at the last condense the whole with foot-shaped pluggers and finish in the usual way.

In the fourth class, *viz.*, proximals between bicuspids and molars, if the grinding surface is not weakened or gone, I pursue the same plan as in the incisors; but, if a compound cavity, I depend for retention on the lateral walls as well as the cervical, inserting the cylinders with the end looking toward the adjoining tooth, letting it protrude, making my basket as usual, and finishing with cohesive gold, condensing thoroughly wherever the soft gold came to the surface, and finishing with disks and tape.

In the fifth and last class referred to, those on the labial surfaces of incisors and cuspids, and on the buccal surfaces of molars, it has been my practice to bevel the edges as far as practicable throughout the whole margin, making an undercut all the way around with wheel-shaped burs, and inserting a cylinder on end, one that would protrude from a third to half its length beyond the cavity, and then make a cavity in the cylinder, forcing the soft gold into the undercut and filling the cavity thus formed with cohesive foil, and at last condensing the whole with foot-shaped points (as it were to head down a rivet) finishing with stones, etc.

One danger to the beginner in this method, is that the cylinders will not be of a proper size. They will either be too large to be inserted, or so small they will not be retained in place.

The one great object to be attained in any filling, except the restoration of contour, is to perfectly adapt the gold to the margins of the cavity; and there is no one that will attempt to gainsay the fact that the softer the gold the more easily it can be adapted to the walls and edges, and after the filling is inserted, the protruding ends of the cylinder can be malleted or burnished down with far less danger to the edges of the cavity. No operation is stronger than its weakest part, and generally the danger line is at the cervical border. In fillings inserted wholly of cohesive gold the trouble is not in regard to their coming out, but of not making a perfect and tight joint at the cervical and lingual borders.

All fillings are in a measure like a rivet, and there can be no better comparison than that all iron-workers use a rivet of as soft iron as is possible, consistent with the strength required. A steel rivet, though much stronger, does not adapt itself nearly as easily and well as one of soft iron; and, as one writer on cohesive and non-cohesive gold expresses himself in regard to burnishing cohesive gold to adapt it to the borders of the cavity, "one might as well try to burnish down a piece of cast iron.— *I. C. Curtis in Dental Cosmos.*

